# Membrane-Type-3 Matrix Metalloproteinase (MT3-MMP) Functions as a Matrix Composition-Dependent Effector of Melanoma Cell Invasion

**DOI:** 10.1371/journal.pone.0028325

**Published:** 2011-12-02

**Authors:** Olga Tatti, Mariliina Arjama, Annamari Ranki, Stephen J. Weiss, Jorma Keski-Oja, Kaisa Lehti

**Affiliations:** 1 Research Programs Unit, Molecular Cancer Biology, University of Helsinki, Helsinki, Finland; 2 Departments of Pathology and Virology, Haartman Institute, Helsinki University Central Hospital, Helsinki, Finland; 3 Department of Dermatology and Allergology, Skin and Allergy Hospital, Helsinki University Central Hospital, Helsinki, Finland; 4 Division of Molecular Medicine and Genetics, Life Sciences Institute, University of Michigan, Ann Arbor, Michigan, United States of America; 5 Research Programs Unit, Genome-Scale Biology, Biomedicum Helsinki, University of Helsinki, Helsinki, Finland; The University of Hong Kong, Hong Kong

## Abstract

In primary human melanoma, the membrane-type matrix metalloproteinase, MT3-MMP, is overexpressed in the most aggressive nodular-type tumors. Unlike MT1-MMP and MT2-MMP, which promote cell invasion through basement membranes and collagen type I-rich tissues, the function of MT3-MMP in tumor progression remains unclear. Here, we demonstrate that MT3-MMP inhibits MT1-MMP-driven melanoma cell invasion in three-dimensional collagen, while yielding an altered, yet MT1-MMP-dependent, form of expansive growth behavior that phenocopies the formation of nodular cell colonies. In melanoma cell lines originating from advanced primary or metastatic lesions, endogenous MT3-MMP expression was associated with limited collagen-invasive potential. In the cell lines with highest MT3-MMP expression relative to MT1-MMP, collagen-invasive activity was increased following stable MT3-MMP gene silencing. Consistently, MT3-MMP overexpression in cells derived from less advanced superficially spreading melanoma lesions, or in the MT3-MMP knockdown cells, reduced MT1-MMP-dependent collagen invasion. Rather than altering MT1-MMP transcription, MT3-MMP interacted with MT1-MMP in membrane complexes and reduced its cell surface expression. By contrast, as a potent fibrinolytic enzyme, MT3-MMP induced efficient invasion of the cells in fibrin, a provisional matrix component frequently found at tumor-host tissue interfaces and perivascular spaces of melanoma. Since MT3-MMP was significantly upregulated in biopsies of human melanoma metastases, these results identify MT3-MMP as a matrix-dependent modifier of the invasive tumor cell functions during melanoma progression.

## Introduction

Cancer-related mortality is generally associated with the development of metastatic lesions. During the progression to metastatic cancer, tumor cells invade through extracellular matrix (ECM) barriers such as the basement membrane and interstitial stroma, enter vascular or lymphatic vessels, extravasate, and colonize distant organs [1], [2]. Comparisons between the gene expression profiles of non-metastatic and metastatic cancers have revealed distinct genetic footprints for each disease state [3]. In such analyses, the proteolytic enzyme, membrane-type matrix metalloproteinase 1 (MT1-MMP, MMP14), is often linked to the metastatic disease [4], [5] with the protease upregulated in tumor cells as well as surrounding stromal cells [6]–[9]. The strongest MT1-MMP induction often correlates with the transition of tumor cells to a rapidly invasive mesenchymal phenotype [10], and its elevated expression in cancer tissues correlates frequently with disease aggressiveness and poor prognosis [5], [7].

The MT-MMP family comprises six members, MT(1–6)-MMPs. Each of the proteases have been implicated in tumor progression, although their expression pattern and known functions appear distinct [11]–[14]. MT1- and MT2-MMP (MMP15) can each drive tumor cell invasion through basement membranes and the collagen type I-rich interstitial stroma [10], [15], while MT3-MMP (MMP16) cannot efficiently cleave native collagen type I or confer cells with collagen-invasive ability *in vitro* or *in vivo* [15]–[17]. However, when overexpressed, both MT1- and MT3-MMP mediate cell invasion in cross-linked fibrin gel, a provisional form of extracellular matrix commonly deposited within tumor tissues and perivascular spaces *in vivo* [18]. Unlike wide MT1-MMP expression in different cancers, notable MT3-MMP mRNA levels have been detected in relatively few types of cancer, such as gliomas, hepatocellular carcinoma, gastric cancer and melanoma, where its translation efficiency, protein expression and function remain poorly defined [11], [19]–[22].

Metastatic melanoma has a poor prognosis with a 5-year survival rate ranging between 5–10% due to its resistance to available cancer therapies [23]. As such, understanding the molecular mechanisms that underlie melanoma metastasis is essential for the development of new prognostic indicators as well as strategies for disease treatment. Consistent with the central role played by MT1-MMP in mediating the pericellular ECM degradation necessary for cell invasion, the expression of the protease has been linked to the development of metastatic lesions in melanoma [4]. Interestingly, MT3-MMP is specifically upregulated in nodular melanoma, the most aggressive melanoma type, comprising about 15% of all melanoma cases [24], [25]. Unlike the more common, superficially spreading form of melanoma that is characterized by a pattern of slow radial growth eventually followed by vertical growth phase and metastasis, nodular melanomas grow rapidly in thickness, and are often metastasized at the time of diagnosis [26]. As the functional contribution of MT3-MMP in melanoma progression has remained undefined, we have here examined its function using gene-silencing and overexpression in different types of melanoma cells. The invasive and growth properties of these cells were defined within the confines of the three-dimensional (3D) type I collagen or fibrin matrices that typify the surrounding host ECM environment.

## Results

### MT3-MMP is overexpressed in human melanoma metastases and metastatic melanoma cell lines

**Figure 1:**
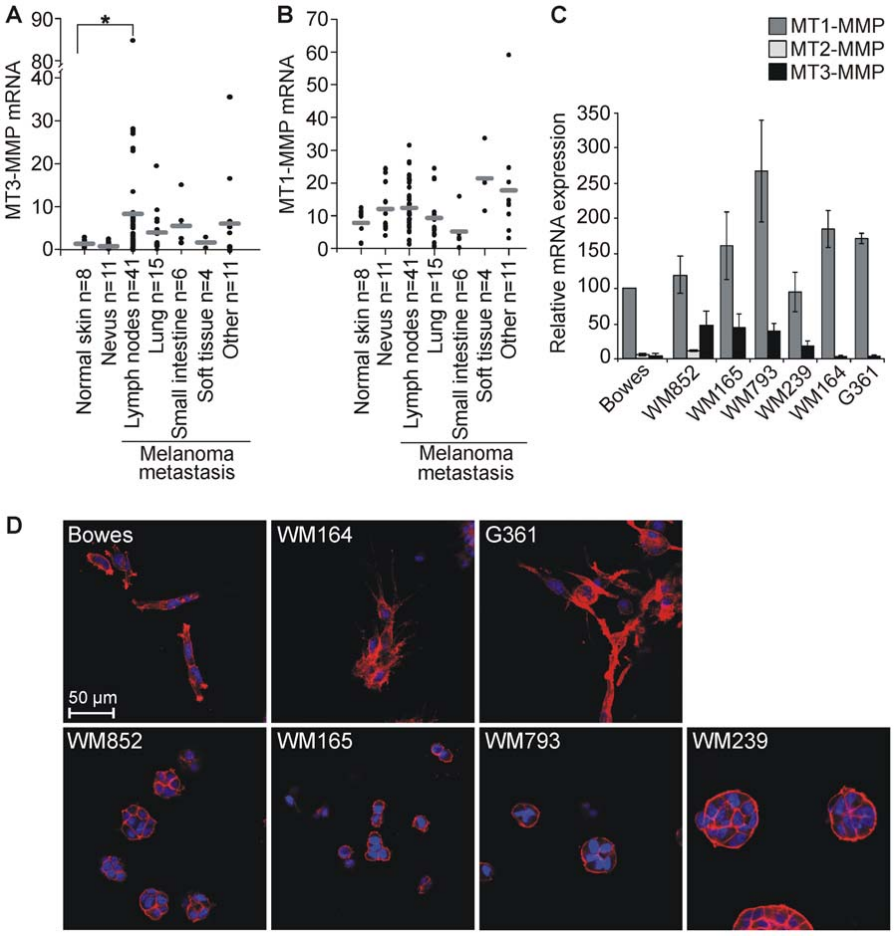
MT3-MMP is expressed in melanoma lymph node metastases and metastatic cell lines. MT3-MMP (A) and MT1-MMP (B) mRNA expression levels in human tissue biopsies from normal skin (n = 8), nevus (n = 11), and melanoma metastases (n = 77) were assessed by qPCR. cDNAs were normalized against beta-actin. Soft tissue comprise metastases obtained from subcutaneous tissue of groin (n = 2), pelvis (n = 1) and thigh (n = 1). Other organs comprise single or double biopsies from melanoma metastases to skin, stomach, liver, mediastinum, ovary, kidney and brain. *p = 0.028 (p = 0.031 if the highest outlying lymph node metastasis value of 84.4 is excluded). (C) Relative values of average MT1-, MT2- and MT3-MMP mRNA expression were analyzed by qPCR in 7 melanoma cell lines as indicated. (D) The melanoma cells were embedded inside 3D collagen type I gels. After 4-d culture, the 3D cell/matrix constructs were fixed and stained with TRITC-phalloidin (red) and DAPI (blue). Fluorescence micrographs of the collagen gels show representative cell colonies.

MT1-MMP is frequently overexpressed in different types of melanoma [4], [27], whereas mean MT3-MMP expression is not increased in primary melanoma compared to normal skin (GeneSapiens, www.genesapiens.org; [28]). However, increased levels of MT3-MMP are associated with the most aggressive nodular melanoma [24], [25]. To assess if MT3-MMP expression is more generally linked to melanoma progression, we analyzed MT1-MMP and MT3-MMP mRNA expression in human tissue biopsies of normal skin (n = 8), benign nevi (n = 11), and melanoma metastases (n = 77). Significantly, MT3-MMP was >8-fold upregulated in the lymph node metastases as compared to normal skin (p = 0.028; Fig. 1A) whereas low MT3-MMP expression observed in benign nevi was comparable to normal skin (Fig. 1A). MT3-MMP was also upregulated in melanoma metastases to lung (∼4-fold), small intestine (∼6-fold) and in a single tissue sample of brain metastasis (∼4.5-fold). In contrast, notable MT1-MMP mRNA levels were detected in several of the normal skin samples, and the slightly increased expression in both lymph node metastases and benign nevi were not statistically significant (Fig. 1B).

Furthermore, four different metastatic melanoma cell lines tested (WM852, WM164, WM165 and WM239) as well as the Bowes, G361 and WM793 cells derived from non-metastatic primary melanoma all expressed MT1-MMP, whereas MT3-MMP was expressed in three cell lines originally isolated from metastatic melanomas (WM852, WM165 and WM239) and WM793 cell line isolated from advanced primary melanoma (Fig. 1C). To investigate the growth and invasion of these cells, they were implanted as single cells in cross-linked collagen matrix that typifies the surrounding ECM environment in collagen-rich skin. Under these conditions, MT1-MMP expression remained high or was even enhanced as compared to cells in monolayer culture or inside a 3D fibrin matrix (Fig. S1). Significant MT3-MMP expression remained unaltered and restricted to the same cells in the monolayer and 3D cultures (Fig. S1). During a 4-d culture within collagen, Bowes, G361 and WM164 cells all devoid of significant MT3-MMP expression grew and invaded efficiently in elongated morphology (Fig. 1D, upper panel). In contrast, the MT3-MMP expressing cells grew as either non-invasive sphere-shaped colonies or groups of rounded cells within collagen (Fig. 1D, lower panel), suggesting that MT3-MMP could have an effect on melanoma cell invasion.

### MT3-MMP expression is associated with rapid fibrin invasion and poor collagen invasion

**Figure 2:**
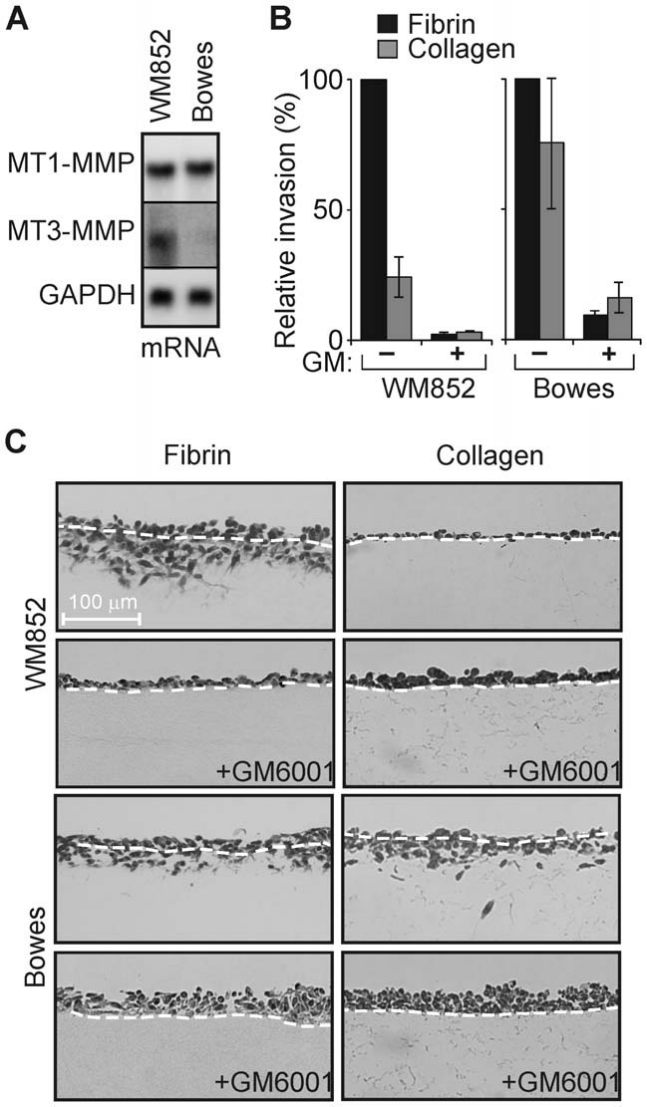
MT3-MMP expressing WM852 melanoma cells invade readily fibrin but have limited ability to invade collagen type I. (A) MT1- and MT3-MMP mRNA expression in WM852 cells from nodular melanoma and Bowes cells from superficially spreading melanoma were analyzed by Northern blotting. GAPDH mRNA levels served as an internal standard. (B) The cells were allowed to invade 3D fibrin and type I collagen for 7 d. Quantitative results are expressed as the number of invasive cells per microscopic field (n = 3). The number of invasive cells in fibrin was set to 100%. (C) Light micrographs of fibrin and collagen cross-sections visualize the invasion of WM852 and Bowes cells. GM6001 (10 µM) was added where indicated. White dotted lines mark the surface of the matrix.

WM852 cells, isolated from the metastatic lesions of nodular melanoma, and Bowes cells, derived from early stage superficially spreading melanoma [29]–[31], were used to begin characterizing the functions of MT1-MMP and MT3-MMP in melanoma. These cells expressed MT1-MMP at comparable levels (Fig. 1C and 2A). By contrast, only WM852 cells expressed significant levels of MT3-MMP (Fig. 1C and 2A), while neither Bowes nor WM852 cells expressed MT2-MMP (Fig. 1C). To asses the relative invasive activities of the cells, they were cultured atop 3D gels of type I collagen, or fibrin, which deposition is enhanced at perivascular sites as well as tumor-host interfaces of melanoma [32]. During a 7-d culture period, WM852 cells efficiently invaded fibrin gels, but displayed only minor collagen-invasive activity (Fig. 2B and C). By contrast, Bowes cells readily invaded both collagen and fibrin matrices (Fig. 2B and C). In accordance with previously described functions for MT-MMPs in cell invasion [15], [18], [33], the ability of either Bowes or WM852 cells to infiltrate collagen or fibrin gels was blocked completely by the synthetic MMP-inhibitor, GM6001 (Fig. 2B and C). Taken together, these results suggest that MT3-MMP expression is compatible with rapid tumor cell invasion into fibrin, whereas in WM852 cells it was associated with the limited ability of MT1-MMP to support collagen invasion.

### MT3-MMP knockdown enhances WM852 melanoma cell invasion into collagen

**Figure 3:**
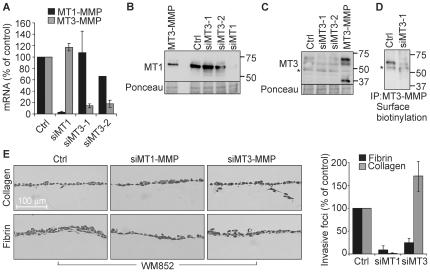
MT3-MMP silencing inhibits fibrin invasion and enhances collagen invasion of WM852 cells. (A) MT1-MMP and MT3-MMP mRNA expression in WM852 cells transfected with control siRNA (Ctrl) or siRNAs targeting MT1-MMP (siMT1) or MT3-MMP (siMT3-1 and siMT3-2) were quantified by qPCR (n = 3). (B and C) The protein levels of MT1-MMP (B) and MT3-MMP (C) were assessed by immunoblotting after transient transfection of the siRNAs or MT3-MMP cDNA (MT3-MMP) as indicated. MT1-MMP was detected as a 60 kDa band and MT3-MMP as 65/60 kDa bands that were downregulated by the corresponding siRNAs. An additional MT3-MMP fragment of 37 kDa in size was detected in the cells after transient transfection with MT3-MMP cDNA. Ponceau-staining served as a loading control. Asterix marks a non-specific band. (D) Cell surface MT3-MMP was detected by surface biotinylation of cells transfected with control siRNA or the most efficient MT3-MMP siRNA (siMT3-1), followed by immunoprecipitation. Asterix marks a non-specific band. (E) Light micrographs of fibrin and collagen cross-sections visualize the invasion of WM852 cells transfected with the indicated siRNAs. The cells were plated atop 3D fibrin and type I collagen 1 d after siRNA transfections and allowed to invade for 5 d. Quantitative results are expressed as the number of invasive foci per microscopic field (n = 3).

To define the effects of MT3-MMP on the melanoma cell invasion, we assessed the impact of silencing endogenous MT1-MMP or MT3-MMP expression by specific siRNAs in WM852 cells (over 80% silencing efficiencies by qPCR; Fig. 3A). The down-regulation of MT1-MMP or MT3-MMP protein was confirmed by immunoblotting (Fig. 3B and C). MT3-MMP, which was detected as the major 65 kDa protein in both control cells expressing endogenous protease and cells transiently transfected with MT3-MMP cDNA, was markedly suppressed by MT3-MMP siRNAs (Fig. 3C). Likewise, endogenous MT3-MMP detected by surface biotinylation followed by MT3-MMP immunoprecipitation in control WM852 cells was barely detectable after transfection with the most efficient MT3-MMP siRNA (Fig. 3D). When plated atop the collagen matrices, the control siRNA-transfected cells displayed only minor invasive potential during a 5-d assay, which was completely abrogated by MT1-MMP silencing as expected (Fig. 3E). By contrast, MT3-MMP knockdown unexpectedly enhanced collagen invasion (1.7±0.3 fold, n = 3, p = 0.05), whereas fibrin invasion was inhibited by silencing either MT1-MMP or MT3-MMP (Fig. 3E).

### MT3-MMP facilitates Bowes melanoma cell invasion into fibrin but restricts collagen invasion

**Figure 4:**
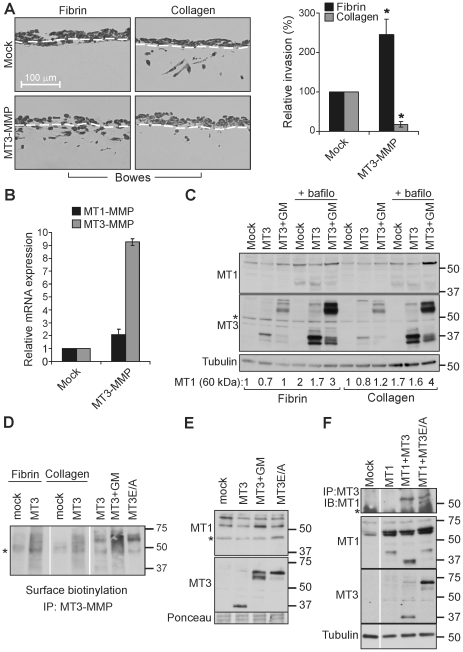
MT3-MMP induces fibrin invasion but inhibits collagen invasion of Bowes melanoma cells. (A) The stable cell pools transfected with MT3-MMP cDNA (MT3-MMP) or mock vector (Mock) were allowed to invade 3D fibrin or collagen gels for 5 d. Quantitative results are expressed as the number of invasive cells per microscopic field relative to the mock-transfected cells (n = 3, *p = 0.02). White dotted lines in the representative light micrographs of fibrin and collagen cross-sections mark the level below which the invaded cells were counted. (B) Chart shows average MT1-MMP and MT3-MMP mRNA expression relative to mock-transfected cells as assessed by qPCR. (C) The stable mock or MT3-MMP expressing cells were treated with GM6001 (10 µM) and bafilomycin A (100 nM) for 16 h as indicated, followed by MT3-MMP and MT1-MMP detection by immunoblotting. Full-length and processed forms of MT3-MMP were detected as 65/60 kDa and 37 kDa bands, respectively, and MT1-MMP as 60 kDa and 37–43 kDa bands. Tubulin served as a loading control. Asterix marks a non-specific band. (D) Cell surface MT3-MMP was detected by surface biotinylation of the stable cells on fibrin and collagen or cells transfected with cDNA for MT3-MMP or catalytically inactive MT3-MMP (MT3E/A) followed by MT3-MMP immunoprecipitation and detection with peroxidase-conjugated streptavidin. Cells were treated with GM6001 (10 µM) for 16 h prior to surface biotinylation where indicated. Asterix marks a non-specific band. (E–F) Bowes cells expressing the indicated cDNAs were lysed, followed by immunoblotting or immunoprecipitation. GM6001 (GM, 10 µM) was added where indicated. Ponceau staining and tubulin served as a loading control. Asterix marks non-specific bands.

Given the increased collagen-invasive activity of WM852 cells after MT3-MMP silencing, we next sought to define the effects of stable MT3-MMP expression on the invasion of Bowes melanoma cells, which normally display almost undetectable levels of the transcript. Stable transfectants increased MT3-MMP mRNA expression ∼9-fold (a level ∼50% lower than endogenous expression in WM852 cells) while stimulating fibrin invasion 2.5±0.3 fold (n = 3; p = 0.02; Fig. 4A and B). Importantly, increasing MT3-MMP expression markedly reduced collagen invasion by ∼80% (0.17±0.07, n = 3; p = 0.021; Fig. 4A), thus shifting the invasive phenotype of Bowes cells towards that displayed by WM852 cells.

In these stable Bowes cells, MT3-MMP was detected predominantly as a ∼37 kDa cleaved fragment by immunoblotting (Fig. 4C), and GM6001 enhanced the levels of 60–65 kDa proteins that most likely correspond to the full-length and activated or differentially glycosylated forms of MT3-MMP protein (Fig. 4C; [34]). After inhibition of lysosomal degradation by bafilomycin A, mostly the cleaved 37-35 kDa fragments of MT3-MMP were increased (Fig. 4C). Nevertheless, the major MT3-MMP form exposed to biotinylation on the surface of Bowes cells was the full-length/activated MT3-MMP, whereas only a faint band corresponding to the 37 kDa fragment was detectable by surface biotinylation (Fig. 4D). Notably, the cleavage to 37-35 kDa fragments was inhibited in tandem with dramatically increased full-length/activated MT3-MMP by simultaneous treatment with GM6001 and bafilomycin A (Fig. 4C). Since both GM6001 and the inactivating E247A mutation (MT3E/A, Fig. S2) that stabilized the 60–65 kDa MT3-MMP also enhanced the levels of MT3-MMP on the cell surface (Fig. 4D and E), these results suggest that constitutive autocatalytic processing of the cell surface MT3-MMP followed by internalization and lysosomal degradation of the membrane-bound fragments resulted in unexpectedly rapid MT3-MMP turn-over.

MT3-MMP also slightly reduced the levels of endogenous MT1-MMP protein in cells cultured on fibrin by ∼26% (0.74±0.2), which was reversed by the MMP inhibitor GM6001 (1.14±0.2; Fig. 3C). Likewise, MT3-MMP enhanced the cleavage of MT1-MMP to inactive 37–43 kDa fragments, which were observed after bafilomycin treatment (1.7±0.2 -fold increased processed/total ratio relative to mock; Fig. 4C). Moreover, MT3-MMP, but not MT3E/A, induced the processing of overexpressed MT1-MMP mainly to the ∼37 kDa fragment after transient co-transfection (Fig. 4F). This raised the possibility of *in trans* processing of the MT-MMPs by a mechanism that would resemble the autocatalytic cleavages in MT1-MMP homo-dimers or oligomers [35], [36]. Since such processing would require close physical proximity or interactions, co-immunoprecipitation analyses were carried out. Consistently with an MT1-MMP cleavage by MT3-MMP, MT1-MMP was co-immunoprecipitated in the same complexes with MT3-MMP and MT3E/A (Fig. 4F). However, MT1-MMP expression was less affected by MT3-MMP in cells cultured on collagen (∼20% reduction of 60 kDa MT1-MMP; 1.4±0.2 -fold increased processed/total ratio), suggesting that the regulation of MT1-MMP protein levels may not be the only mechanism how MT3-MMP alters collagen invasion.

### MT3-MMP reduces cell surface MT1-MMP and functions as a matrix composition-dependent effector of cell invasion

**Figure 5:**
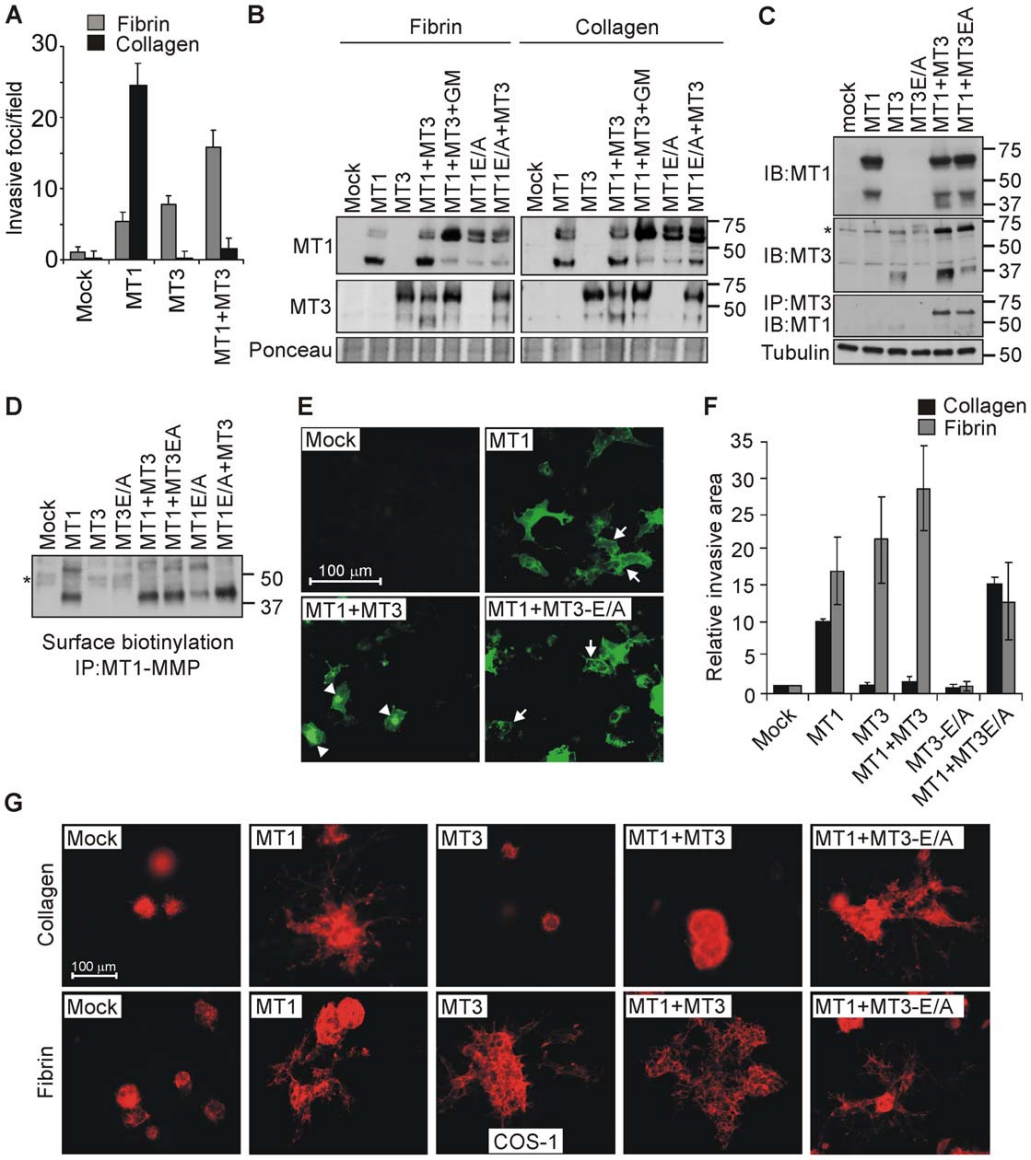
MT3-MMP regulates cell invasion in a matrix-composition dependent manner by driving fibrin invasion, while reducing the cell surface levels and collagen invasive activity of MT1-MMP. (A) COS-1 cells transiently transfected to express MT1-MMP (MT1), MT3-MMP (MT3), or both in combination, were allowed to invade 3D fibrin or collagen gels for 5 d. Quantitative results are expressed as the number of invasive foci per microscopic field (n = 3). (B–C) MT1-MMP, inactive MT1-MMP (MT1E/A), MT3-MMP, and MT3E/A were expressed alone or in different combinations in COS-1 cells for 48 h followed by immunoblotting and immunoprecipitation as indicated. GM6001 (GM, 10 µM) was added where indicated. Ponceau-staining and tubulin served as loading controls (n = 3). Asterix marks a non-specific band. (D) Cell surface MT1-MMP was detected from the transfected cells by surface biotinylation followed by immunoprecipitation. Asterix marks a non-specific band. (E) The transfected cells were fixed and stained for MT1-MMP with an antibody against its catalytic domain. Arrows indicate MT1-MMP staining on the cell surface in the cells expressing MT1-MMP alone or with inactive MT3E/A, as opposed to the perinuclear staining (arrowheads) in the cells co-expressing MT1-MMP and MT3-MMP. (F) The transfected cells were embedded in 3D collagen or fibrin as single cell suspension and allowed to grow for 5 d. Relative sizes of invasive areas per colony were calculated using ImageJ software (n = 3). (G) Fluorescence micrographs visualize representative colonies of COS-1 cells cultured inside collagen or fibrin and stained for filamentous actin.

To define the interrelated functions of MT1-MMP and MT3-MMP in cell invasion, COS-1 cells, which are largely devoid of endogenous MMPs, were engineered to express MT1-MMP, MT3-MMP, or both proteases in combination. Consistent with the fact that both MT1-MMP and MT3-MMP can degrade fibrin [18], either MT1-MMP or MT3-MMP conferred the cells with the ability to invade fibrin gels and displayed additive effects when co-expressed (Fig. 5A). In contrast, while MT1-MMP alone induced COS-1 cell invasion into collagen matrices, MT3-MMP, which is ineffective type I collagenase, suppressed MT1-MMP-dependent collagen invasion upon co-expression (Fig. 5A), a result consistent with the conclusion that MT3-MMP interfered with the collagen-invasive activity of MT1-MMP in melanoma cells.

In COS-1 cells cultured on either fibrin or collagen the effects of MT3-MMP co-expression on total MT1-MMP protein levels were minor (Fig. 5B). In these co-transfected cells, MT1-MMP instead reduced the levels of full-length MT3-MMP with a concomitant increase in the processed, 37 kDa fragment of the protease via a process that was blocked by GM6001 (Fig. 5B). The catalytically-inactive MT1E/A did not induce MT3-MMP processing (Fig. 5B), indicating that the increased MT3-MMP cleavage required MT1-MMP activity. Furthermore, MT3-MMP slightly enhanced the processing of MT1E/A in the cells cultured on collagen (Fig. 5B). Consistent with the interaction in Bowes cells, both MT3-MMP and MT3E/A were co-precipitated with MT1-MMP (Fig. 5C). Interestingly, when 3-fold higher amount of cDNA for MT3-MMP than for MT1-MMP was transfected, MT1-MMP processing to 37–43 kDa fragments was increased also in COS-1 cells (Fig. 5C) suggesting that a dose-dependent competition may at least partially dictate the order of the cleavages between these MT-MMPs. Furthermore, even after equal transfection of the MT1-MMP and MT3-MMP cDNAs, MT3-MMP notably enhanced the processing of the fraction of MT1-MMP and MT1E/A exposed to cell surface biotinylation (Fig. 5D). Accordingly, MT3-MMP, but not the catalytically-inactive MT3E/A, decreased cell surface MT1-MMP in tandem with intracellular MT1-MMP accumulation as detected by immunofluorescence (Fig. 5E and S3).

Since MT1-MMP surface levels can affect 3D growth and invasion pattern of the cells [37]–[39], we assessed next the impact of MT1-MMP and MT3-MMP on 3D cell growth and mode of invasion. As such, COS-1 cells transiently transfected to express MT1-MMP, MT3-MMP, catalytically-inactive MT3E/A, or MT1-MMP and MT3-MMP in combination, were embedded as single cells in either type I collagen or fibrin gels. During a 5-d culture period, mock-transfected cells formed small colonies which were not invasive in either matrix (Fig. 5F and G). As expected, MT3-MMP-expressing cells invaded only in fibrin (21.6±6.0 -fold increase in invasive area compared to mock cells, with a negligible increase in collagen invasion), whereas MT1-MMP-expressing cells generated both invasive colonies characterized by multicellular sprouts as well as clusters of cells invading in a single-cell fashion in either collagen or fibrin gels (in collagen, 9.9±0.4 –fold increase in invasive area as compared to mock-transfected cells, and in fibrin, 17.0±4.6 –fold increase compared to mock-transfected cells; Fig. 5F and G). Interestingly, the co-expression of MT3-MMP with MT1-MMP resulted in weakly sprouting or sphere-shaped colonies in collagen (Fig. 5F and G), while increasing fibrin-invasive activity of the cells (28.5±5.9 –fold increase compared to mock-transfected cells; Fig. 5F and G). By contrast, the expression of MT3E/A did not inhibit collagen invasion or induce fibrin invasion (Fig. 5F and G). These results indicate that MT3-MMP-dependent proteolytic activity promoted fibrin invasion, but resulted in decreased MT1-MMP surface levels and inhibition of collagen invasion.

### MT3-MMP enhances fibrin invasion and MT1-MMP-dependent nodular-type cell growth in 3D collagen

**Figure 6:**
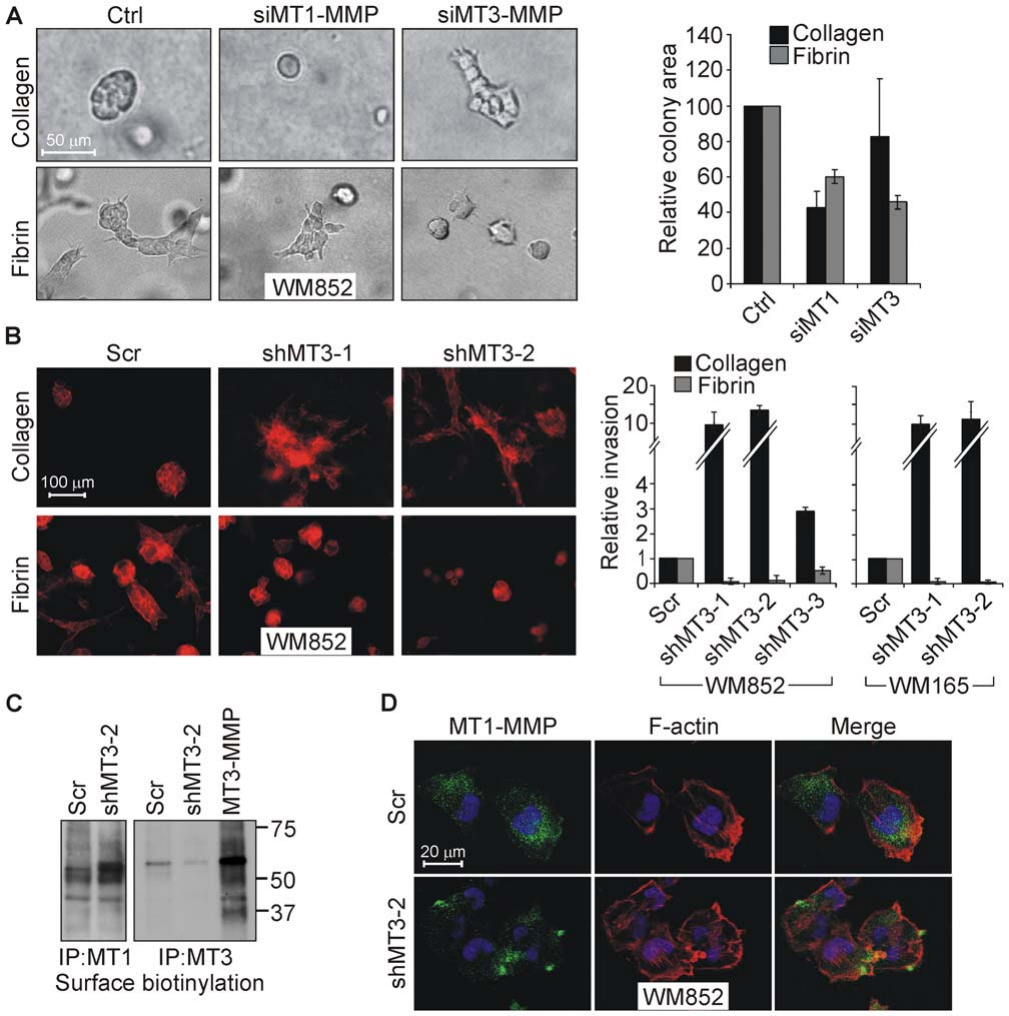
MT3-MMP promotes invasion in 3D fibrin and adhesive, nodular-type cell growth in 3D collagen. (A) WM852 cells transfected with the siRNAs against MT1-MMP (siMT1-MMP) and MT3-MMP (siMT3-MMP) were embedded in 3D collagen type I or fibrin gels as single cell suspension. Light micrographs show representative cell colonies that were formed after 5-d assay. Chart on the right shows the relative sizes of the cell colonies (n = 3). (B) Stable WM852 and WM165 cell pools expressing control scrambled shRNA (scr) or MT3-MMP targeting shRNAs (shMT3-1, shMT3-2 and shMT3-3) were allowed to grow inside 3D collagen or fibrin gels for 14 d. The fixed 3D cultures were stained with TRITC-phalloidin and photographed (see Fig. S4D for representative micrographs of WM165 cell colonies). Chart shows relative areas of invasive sprouts. (C) Cell surface MT1-MMP and MT3-MMP were detected by surface biotinylation of stable WM852 cells expressing the most potent shRNA against MT3-MMP (shMT3-2) or scrambled shRNA (Scr), followed by immunoprecipitation using MT1-MMP and MT3-MMP antibodies. WM852 cells after transient transfection with MT3-MMP cDNA (MT3-MMP) were used as a control for the immunoprecipitation of endogenous MT3-MMP. (D) Epifluorescence micrographs visualize the increased MT1-MMP (green) on the surface of shMT3-2 expressing cells. The cells were counterstained with DAPI for nuclei (blue) and phalloidin for filamentous actin (red).

To assess next the impact of the endogenous proteases on melanoma cell growth and pattern of invasion, WM852 cells were cultured within 3D collagen gel. The control cells grew as compact nodular cell colonies, while in 3D fibrin the cells formed invasive colonies with multicellular sprouts (Fig. 6A and B). As expected, 3D growth in collagen was blocked when MT1-MMP expression was silenced, a result consistent with previous reports (Fig. 6A; [37]). Remarkably, following MT3-MMP knockdown by siRNAs or by lentiviral shRNAs (Fig. S4A), the cell colonies in collagen became irregular in outline and displayed invasive activity, while the average size of the colonies was less affected (Fig. 6A and B, Fig. S4B). Concomitantly with increased collagen invasion, MT1-MMP levels on the cell surface were increased after MT3-MMP knockdown, as assessed by surface biotinylation and immunofluorescence (6C and D), while MT1-MMP mRNA remained unaffected (Fig. S4A). In contrast, efficient MT3-MMP silencing by shMT3-1 and shMT3-2 significantly reduced both colony size and invasive sprout formation in 3D fibrin (Fig. 6A and B). These changes correlated with the dose of MT3-MMP, as the modest MT3-MMP knockdown by shMT3-3 had only minor effects on the matrix composition dependent 3D growth and invasion (Fig. 6B and S4A). Likewise, stable MT3-MMP knockdown in the MT3-MMP expressing WM165 melanoma cells (Fig. S4C) led to increased cell elongation and invasive sprouting of cell colonies in collagen (Fig. 6B and S4D). Unexpectedly, MT3-MMP knockdown reduced the invasion of WM852 cells in fibrin even more efficiently than MT1-MMP knockdown (Fig. 6A; relative colony size 0.60±0.04, and invasion 0.41±0.1 for siMT1-MMP transfected cells; size 0.46±0.04, and invasion 0.20±0.02 for siMT3-MMP transfected cells, n = 3). MT3-MMP silencing also dramatically reduced the invasive growth of WM165 cell colonies in fibrin (Fig. 6B and S4D). In contrast, the invasion and growth of WM793 cells with higher MT1-MMP to MT3-MMP expression ratio was only slightly affected in 3D collagen by MT3-MMP knockdown (Fig. 1, S4E and S4F). The MT3-MMP knockdown also did not alter growth or invasion of Bowes cells in 3D collagen or fibrin, consistent with the low expression levels of endogenous MT3-MMP in these cells (Fig. S5A–D).

**Figure 7:**
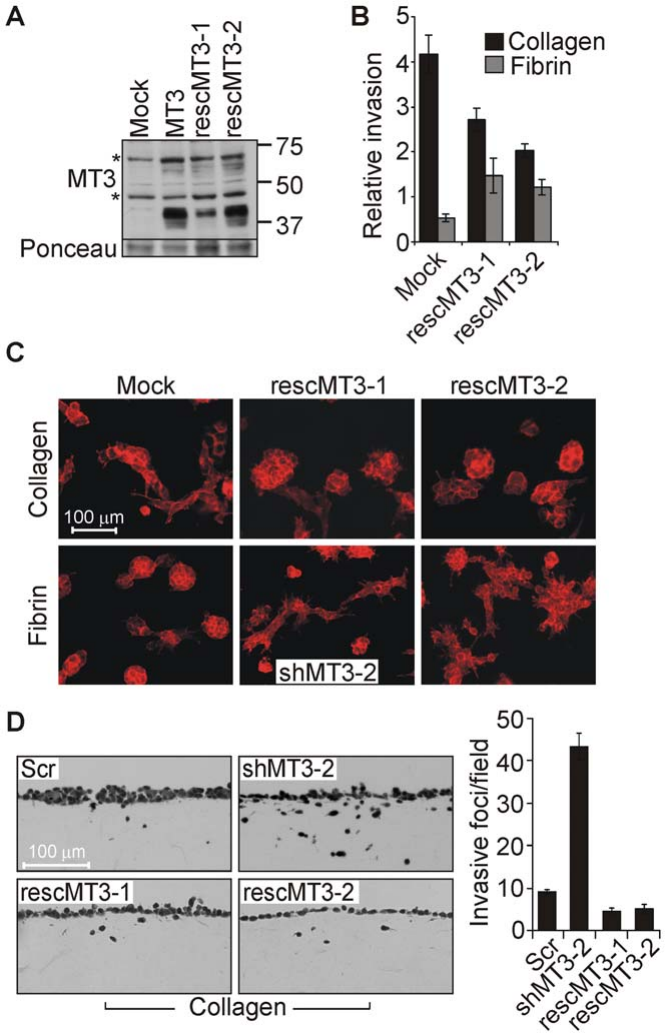
Transient MT3-MMP expression in the MT3-MMP knockdown cells rescues nodular-type growth in collagen in conjunction with increased fibrin invasion. (A) MT3-MMP was detected by immunoblotting in COS-1 cells transiently expressing mock vector (Mock), MT3-MMP (MT3), or rescue constructs rescMT3-1 and rescMT3-2. Ponceau staining served as a loading control. Asterix marks non-specific bands. (B) WM852 cells stably expressing MT3-MMP targeting shRNA (shMT3-2) transfected to express MT3-MMP rescue vectors (rescMT3-1 and rescMT3-2) or mock vector were embedded in 3D collagen type I and fibrin gels. Chart shows relative percentage of invasive colonies formed after 5 d, n = 3. (C) Fluorescent micrographs show representative colonies stained for F-actin (red) to visualize the cells. (D) WM852 cell pools after lentiviral expression of scrambled shRNA (Scr) and shMT3-2 as well as knockdown cells transfected with MT3-MMP rescue vectors (rescMT3-1 and rescMT3-2) were allowed to invade into collagen type I gels for 7 d followed by imaging and quantification, n = 3.

To confirm the specific invasion regulating functions of MT3-MMP in collagen and fibrin matrices, we constructed two MT3-MMP rescue plasmids with silent mutations in the shMT3-2 targeting sequence (rescMT3-1 and rescMT3-2; Fig. 7A). Transient transfection of these constructs in the MT3-MMP knockdown WM852 cells diminished collagen invasion and facilitated fibrin invasion (over 70% transfection efficiency in the shMT3-2 cells using TransIT-2020), indicating that the observed 3D cell phenotypes were indeed MT3-MMP-dependent (Fig. 7B and C). Likewise, the enhanced collagen invasion of the MT3-MMP knockdown cells was reversed to the level seen in scrambled shRNA expressing cells after transient expression of the rescue constructs in the knockdown cells (Fig. 7D). Taken together, these results suggest that MT3-MMP, essential for WM852 and WM165 melanoma cell invasion in fibrin, is a strong negative regulator of melanoma cell invasion within 3D collagen, where it induced adhesive nodular-type growth pattern.

## Discussion

Unlike the strongly pro-invasive MT1-MMP that is widely overexpressed in the aggressive and metastatic tumors, MT3-MMP expression has been reported in only a few tumor types [11], [19]–[21]. These include nodular melanoma, the most aggressive melanoma subtype characterized to date [24], [25]. Consistent with the compact sphere-like growth pattern characteristic for nodular melanoma, we found that WM852 cells, originally derived from a nodular melanoma metastasis, and the other MT3-MMP expressing cell lines from advanced melanomas grew within 3D type I collagen gels as nodular-type adhesive cell colonies. These colonies displayed a limited ability for invasion of the surrounding collagen matrix as single cells or multicellular sprouts. Unexpectedly, this cell phenotype was dependent on MT3-MMP as silencing its expression enhanced MT1-MMP-driven collagen invasion. Since the nodular-type growth phenotype was rescued when MT3-MMP was overexpressed in the knockdown cells, MT3-MMP is not only unable to support type I collagen-invasive activity [16], [17], but also restricts the proinvasive activity of MT1-MMP in our 3D experimental model. By contrast, MT3-MMP – both alone and in combination with MT1-MMP – conferred cells with prominent fibrin-invasive activity.

In addition to invasion, MT1-MMP is required for tumor growth within the confines of cross-linked matrices of type I collagen, a hydrogel whose structural and mechanical characteristics mimic those of most interstitial tissues *in vivo* 33,37. The pattern of tumor cell growth in collagen can occur either invasively or as an expansive, adhesive mass, which may in part depend on MT1-MMP surface levels and activity [38]. In contrast, recombinant MT3-MMP is a poor type I collagenase [16] and unable to drive cell invasion through native polymerized collagen type I matrix [17], [33], although MT3-MMP itself has also been implicated in the remodeling of 3D collagen [40], 41. In our experiments, Bowes cells derived from superficially spreading melanoma invaded rapidly in 3D collagen as single cells as well as multicellular sprouts in an MT1-MMP dependent manner. As such, MT1-MMP activity could support both local superficial spreading in epidermal and upper dermal tissues as well as invasion into deeper portion of the dermis. However, the collagen-invasive phenotype of Bowes cells was reduced following co-expression of catalytically-active MT3-MMP. These observations are not confined to melanoma cells as COS-1 cells expressing MT1-MMP alone were also highly invasive in 3D collagen, while cells expressing both MT1- and MT3-MMP exhibited only limited collagen invasive ability. By restricting single cell invasion and sprouting, MT3-MMP co-expression led to the MT1-MMP-dependent expansion of nodular cells colonies in 3D collagen.

Given the inability of MT3-MMP to degrade native collagen type I directly and the importance of MT1-MMP homodimerization for its activity in collagen degradation [42], we considered the possibility that MT1-MMP/MT3-MMP heterodimers might form a complex that fails to support collagen adhesion or unwinding. However, although both catalytically active and inactive MT3-MMPs co-immunoprecipitated with MT1-MMP, the catalytically-inactive MT3-MMP mutant did not suppress MT1-MMP-mediated collagen invasion. Alternatively, MT3-MMP could suppress collagen invasion by cleaving MT1-MMP to inactive fragments, or indirectly by catalyzing the processing of other co-operative or invasion regulating proteins such as cell surface adhesion receptors and/or their ligands [43], [44]. In both COS-1 and Bowes cells, MT3-MMP overexpression decreased cell surface MT1-MMP concomitantly with increased MT1-MMP processing to the inactive fragments. In contrast, such processing of endogenous MT1-MMP or MT3-MMP was less evident in WM852 cells, which could be at least partially due to the higher expression levels of TIMP2 and TIMP3 that could inhibit and thus stabilize the MT-MMPs in these cells [29]. However, MT1-MMP levels were increased also on the WM852 melanoma cell surface after knockdown of endogenous MT3-MMP. Thus, the reduced MT1-MMP surface localization was a consistent property of the growing, but modestly invasive cell phenotype induced by MT3-MMP in 3D collagen.

Stabilized intratumoral fibrin networks have been implicated in the progression of several human neoplasms, including brain tumors that are almost devoid of type I collagen [45], [46]. Since MT3-MMP expression is upregulated frequently in tumors that arise in collagen-poor tissues ([28], [47]; www.oncomine.org; www.genesapiens.org), and the effects of MT1-MMP and MT3-MMP were additive promoting invasion in fibrin, MT3-MMP expression in primary tumors associated with fibrin accumulation may promote local tumor spread. This hypothesis and the importance of MT3-MMP for fibrin invasion is strongly supported by our finding that silencing endogenous MT3-MMP in two different melanoma cell lines reduced dramatically their ability to invade and form sprouts in 3D fibrin. Although melanoma arises in collagen-rich skin, abundant fibrin accumulation is frequently found in these tumors within perivascular spaces as well as the vascular and lymphatic vessel-rich tumor-host tissue interface [32]. Within these fibrin-rich sites, MT3-MMP-dependent proteolysis could enhance the passage of melanoma cells into lymphatic vessels, where discontinued basement membranes do not form significant barriers for cell invasion [48], [49].

In addition to primary tumor sites, the accumulation of fibrin-rich provisional matrices around the circulating melanoma cell embolus promotes their metastatic capacity in lungs and lymph nodes [50], [51]. As such, MT3-MMP could further accelerate the metastatic process in such environments via its ability to affect pericellular fibrin remodeling. Consistent with these proposed pro-metastatic functions, MT3-MMP expression was increased in biopsies of human melanoma metastasis. While the nodular vs. superficially spreading origin of the biopsied tissues of the metastatic lesions had not been defined, MT3-MMP expression was not restricted to nodular melanoma-derived cell lines. Rather, WM165 and WM239 cells derived from the metastases of superficially spreading melanoma as well as in WM793 cells isolated from vertical growth phase primary melanoma also expressed the protease. In this regard, it is interesting to note that the formation of metastatic foci may also depend on the ability of tumor cells to form adhesive expansively growing colonies [52]. Since MT3-MMP had similar effects in the invasive activities of WM852 cells from nodular melanoma metastases and WM165 cells originated from superficially spreading melanoma metastases, we postulate that the protease may play previously unappreciated role in the metastatic process.

To date, minimal information is available about any functions of endogenous MT3-MMP in tumor cells, and the relevant ECM and non-ECM substrates for MT3-MMP remain poorly defined. As supported by a recent report of Nogo receptor 1 shedding by endogenous MT3-MMP in neurons [53], MT3-MMP may modulate also melanoma cell invasion by cleaving transmembrane or pericellular proteins. Like MT1-MMP, MT3-MMP is also efficient cell surface activator of proMMP2 [21]. However, MMP2 is inefficient in driving cell invasion in collagen or fibrin alone [15], [18]. In addition, current results revealed opposing effects of MT3-MMP on fibrin, that is its own substrate, and on collagen type I, that is a poor substrate for MT3-MMP but is instead efficiently cleaved by MT1-MMP. Since MT3-MMP concomitantly reduced MT1-MMP from the surface and MT1-MMP-dependent collagen invasion, current results suggest that MT3-MMP restricts the proinvasive MT1-MMP activity and cell invasion in type I collagen-rich tissues, thus promoting the expansion of nodular melanoma cell colonies. However, as endogenous MT3-MMP activity remains unaffected by MT1-MMP, its fibrinolytic potential could provide melanoma cells with the means to infiltrate perivascular spaces and remodel the tumor cell surrounding provisional matrices during melanoma progression.

## Materials and Methods

### Antibodies and reagents

The following antibodies were used: rabbit polyclonal antibodies against MT1-MMP cytoplasmic tail [35] or hinge domain (RP1, Triple Point Biologics; Ab6004, Chemicon), mouse monoclonal antibodies against MT1-MMP catalytic domain (LEM2-15/8; Millipore) and hemagglutinin (HA; Covance). Rabbit polyclonal antibodies against MT3-MMP hinge domain were from Abcam (ab38968), or kindly provided by Dr. R. Fridman (Wayne State University, Detroit, USA; Ab318). The MMP inhibitor, GM6001 (Calbiochem), phalloidin-TRITC, collagen type I from rat tail (Sigma-Aldrich), bafilomycin A1 (Calbiochem) and recombinant human HGF (R&D systems) were also used.

### Cell culture

Human Bowes melanoma cell line, derived from radial growth phase superficially spreading melanoma [30], was obtained from Dr D. B. Rifkin (Rockefeller University, New York, USA). The other human melanoma cell lines used have been established at the Wistar Institute (Philadephia, PA; [29]). They were originally derived from primary superficially spreading melanoma (G361 and WM793), nodular melanoma metastases (WM852 and WM164), or metastasis of superficially spreading melanoma (WM165 and WM239; [31], [54], [55]). Melanoma cells were cultivated in Eagle's Minimal Essential Medium (MEM) containing 10% heat-inactivated fetal calf serum (FCS). COS-1 monkey kidney cells were cultured according to the instructions (American Type Culture Collection). To generate stable cell pools, transfected Bowes cells were selected using G418 (400 µg/ml; Calbiochem).

### RNA isolation from human tissues and cultured cells

Total mRNAs were extracted from representative microdissected samples of archival paraffin-embedded biopsies of benign intradermal or compound melanocytic nevi (n = 11) and of adjacent normal skin (n = 2) using High Pure RNA Paraffin Kit (Roche) or from cultured cells using RNeasy Mini kit (Qiagen). The nevus biopsies had been removed for diagnostic purposes and the histological diagnosis had been confirmed by the university hospital dermatopathologist. For RNA isolation from 3D cultures, collagen and fibrin were first digested using collagenase type 2 (Worthington) and trypsin (Sigma-Aldrich), respectively (both 2 mg/ml). The cells were then collected by brief centrifugation and lysed for RNA isolation using the RNeasy Mini kit. Reverse transcription was carried out with Random hexamer primers (Invitrogen) and Superscript III reverse transcriptase (Life Technologies). In addition, TissueScan Melanoma Tissue qPCR Arrays I and II (OriGene Technologies) containing cDNAs from human tissue biopsies of normal skin (n = 6) and melanoma metastases (n = 77) were used for the quantification of the MT-MMP transcripts.

### qPCR

The cDNAs were amplified on GeneAmp 7500 Sequence Detector thermal cycler (Applied Biosystems) using TaqMan Universal PCR Master Mix and validated primers (MT1-MMP; Hs 01037006_gH, MT2-MMP; Hs 00233997_m1, MT3-MMP; Hs 00234676_m1, Applied Biosystems). The expression levels were normalized with mRNA for TATA-binding protein (TBP).

### Cell invasion and growth in collagen

Cell invasion was assessed essentially as described [33], [56]. Briefly, type I collagen (4.8 mg/ml, rat tail) was mixed with equal amount of 2×MEM, and pH was adjusted to ∼7.4 by NaOH. Collagen was cast in the upper chambers of Falcon cell culture inserts in 24 well plates and incubated at 37°C for 1 h to allow gelling. Cross-linked fibrin gels were cast in the inserts by combining 75 µl plasminogen-free human fibrinogen (6 mg/ml; Calbiochem) in Hank's Balanced Salt Solution (HBSS) and 75 µl HBSS (pH 7.4) containing 4 U/ml human thrombin and 400 µg/ml aprotinin (both from Sigma-Aldrich). Cells were seeded atop the matrix in the upper chamber in medium containing 1% FCS. GM6001 (10 µM) was added to both upper and lower chambers when used. HGF (25 ng/ml) and medium containing 10% FCS served as chemoattractants in the lower chambers, with medium changed every third day. After a 5–7 d culture period, the cells were fixed with 4% PFA, and paraffin sections were stained with hematoxylin and eosin (H&E-staining). Sections were photographed, and the invaded cells were counted from 8 random sections of each sample. Each assay was performed in triplicate. For 3D growth/invasion assays, collagen (2.4 mg/ml) and fibrin were prepared as above. 5000 cells were suspended in 40 µl hydrogel, the suspension were transferred to a 24-well plate, and incubated for 1 h at 37°C to allow complete gelling. After 4–16 d incubation in complete growth medium, cultures were fixed and photographed using Axiovert 200 microscope (Carl Zeiss). Alternatively, collagen or fibrin gels were stained with phalloidin-TRITC to visualize filamentous actin, and photographed using Axioplan upright epifluorescence microscope (Carl Zeiss). The invasive areas were calculated from 20 random phase-contrast or epifluorescence images of 6 separate gels (49–163 colonies/transfected cell line, n = 3) using ImageJ software.

### Northern hybridization

Total cellular RNA (10 µg) was fractioned by formaldehyde-agarose gel electrophoresis and transferred to a Zeta probe membrane (Bio-Rad) in 20× SSC (1× SSC: 150 mM NaCl, 15 mM sodium citrate, pH 7.0) and fixed by UV crosslinking. cDNA probes corresponding to full-length MT1-MMP and MT3-MMP cDNA were labeled with [^32^P] dCTP by the random priming method (Amersham Pharmacia Biotech). The membranes were hybridized in ExpressHyb hybridization solution (Clonetech) at 58°C for 16–24 h. Washing was carried out in 0.2× SSC containing 0.1% SDS at 63°C. RNA for glyceraldehyde-3-phosphate dehydrogenase (GAPDH) was used as an internal standard.

### RNA interference and cDNAs

Small interfering RNAs (siRNA) targeting MT1-MMP (5′CAGCGATGAAGTCTTCACTTA′3 and 5′TGGCGGGTGAGGAATAACCAA′3), MT3-MMP (5′CCGCCACATACTGTACTGTAA′3 and 5′AAGCACATCACTTACAGTATA′3) and non-silencing control siRNAs (Qiagen) were transfected using Lipofectamine™ 2000 (Life Technologies). Short hairpin RNA (shRNA) targeting MT3-MMP (shMT3-1, TRCN0000052249; shMT3-2, TRCN0000052250; and shMT3-3, TRCN0000052251) or nontargeting scrambled shRNA (Open Biosystems) in pLKO.1 vector were delivered into the cells using lentiviral transduction as described [57]. For stable shRNA expression cells were selected using puromycin (5 µg/ml). The knockdown efficiency was assessed by quantitative PCR (qPCR) after 48 h. Expression vectors for MT3-MMP, MT1-MMP, MT1-MMP with inactivating E240A point mutation (MT1E/A) and HA-tagged MT1-MMP have been described [17], [18], [58] and were transfected using FuGENE (Roche) or TransIT-2020 (Mirus). Inactive MT3-MMP E247A construct (MT3E/A) was kindly provided by Dr. D. Pei [34]. Rescue constructs for MT3-MMP with double silent mutations in shMT3-2 binding sequence rescMT3-1 (T822C-T825C) and rescMT3-2 (T831C-A836C) were generated with primers (5′CTACAGTGAATTAGAAAATGGCAAACGCGACGTGGATATAACC′3 and 5′GGTTATATCCACGTCGCGTTTGCCATTTTCTAATTCACTGTAT′3) and (5′GGCAAACGTGATGTGGACATCACCATTATTTTTGCATCTGG′3 and 5′CCAGATGCAAAAATAATGGTGATGTCCACATCACGTTTGCC′3), respectively, using QuikChange® Site-Directed Mutagenesis Kit (Stratagene).

### Immunoblotting

Cells were lysed with RIPA buffer (50 mM Tris-HCl pH 7.4, 150 mM NaCl, 1% NP-40, 0.5% NaDOC, 0.1% SDS) containing Complete® protease inhibitor cocktail (Roche). Protein contents of the lysates were determined by bicinchoninic acid protein assay (Sigma-Aldrich). Equivalent quantities of protein were size-fractionated by gradient (4–20%) SDS-PAGE followed by transfer to polyvinylidene difluoride membranes. The non-specific protein binding sites were saturated with 5% non-fat milk in TTBS-buffer (TBS containing 0.05% Tween-20) for 30 min. The membranes were washed with TTBS and incubated with primary antibodies overnight, and with biotinylated secondary antibodies (Dako) for 30 min. After incubation with horseradish peroxidase –conjugated streptavidin for 20 min (GE Healthcare), the bound antibodies were detected using enhanced chemiluminescence (Promega). All experiments were repeated for 2–4 times.

### Immunoprecipitation

Endogenous or recombinant MT1-MMP and MT3-MMP were immunoprecipitated from cell lysates by incubation with specific rabbit polyclonal antibodies for MT3-MMP (against hinge domain; Ab38968) or MT1-MMP (against cytoplasmic tail [35] or hinge region; AB6004, Chemicon) and G protein sepharose (GE Healthcare) for 2 h at 4°C. The sepharose particles were then pelleted by centrifugation and washed extensively with PBS. The proteins were eluted with the sample buffer and separated for immunodetection by SDS-PAGE.

### Cell surface labeling

Cell surface biotinylation was performed as described [35]. Briefly, cells were rinsed twice with PBS and incubated with 0.5 mg/ml biotin (Pierce) in PBS on ice for 1 h. The reaction was terminated by washing 3 times for 10 min with 150 mM glycine/TBS. The cells were then lysed and subjected to immunoprecipitation with rabbit polyclonal MT1-MMP or MT3-MMP antibodies. The immunoprecipitated material was resolved by SDS-PAGE, and detected with horseradish-peroxidase-conjugated streptavidin (Daco).

### Immunofluorescence

The cells on glass coverslips were fixed with 4% PFA followed by staining with the indicated primary antibodies and Alexa Fluor-labeled secondary antibodies (Invitrogen). The coverslips were finally mounted on glass slides using Vectashield anti-fading reagent (Vector Laboratories) and examined using the Axioplan microscope.

### Gelatin zymography

MMP-2 was transiently expressed in COS-1 cells, and the conditioned medium was harvested after 18 h. Aliquots of the conditioned medium were incubated with COS-1 cells overexpressing MT1- and MT3-MMPs for 24 h. Aliquots of conditioned medium were then subjected to the gelatin zymography [57].

### Statistical analysis

All numerical values represent mean ± SD. Statistical significance was determined using the Mann-Whitney test.

## Supporting Information

Figure S1 **MT1-MMP and MT3-MMP expression in melanoma cells cultured within 3D collagen and fibrin.** Relative values of average MT1-MMP (A) and MT3-MMP (B) mRNA expression in indicated melanoma cell lines cultured inside 3D collagen and fibrin gels for 48 h were analyzed by qPCR. MT1-MMP and MT3-MMP mRNA expression in Bowes cells cultured in collagen were set to 100%, n = 3.
(TIF)

Figure S2 **Catalytically inactive MT3-MMP does not activate MMP-2.** Gelatin zymogram shows MT1-MMP-mediated MMP-2 activation by COS-1 cells expressing MT1-MMP, MT3-MMP or MT3E/A. MMP-2: latent (L), intermediate (I) and active (A). After harvesting of conditioned media, the cell lysates were subjected to immunoblotting for MT3-MMP and MT1-MMP.
(TIF)

Figure S3 **MT3-MMP reduces MT1-MMP on the cell surface.** COS-1 cells transiently expressing MT1-MMP or MT1-MMP in combination with MT3-MMP or MT3E/A were fixed and stained with antibodies against the hinge domain (RP1) and catalytic domain (LEM2-15/8) of MT1-MMP. Magnified areas indicated by white boxes are presented below the each image for RP1 staining, and on figure 5E for LEM2-15/8 staining.
(TIF)

Figure S4 **MT3-MMP modulates collagen and fibrin invasion of three different melanoma cell lines.** (A) Average MT1-MMP and MT3-MMP mRNA expression in the WM852 cell pools expressing shRNA against MT3-MMP (shMT3-1, shMT3-2 and shMT3-3) relative to cells expressing scrambled shRNA (Scr). (B) WM852 cells stably expressing indicated shRNAs were embedded in 3D collagen type I or fibrin gels as single cell suspension. Light micrographs show representative cell colonies that were formed after 7-d assay. (C) Average MT-MMP mRNA expression in the WM165 cells expressing indicated shRNAs. (D) WM165 cells expressing indicated shRNAs were cultured inside 3D collagen type I and fibrin for 14 d, after which cultures were fixed and stained for filamentous actin. Quantifications of invasive areas are presented on figure 6B. (E) Average MT-MMP mRNA expression in the WM793 cells stably expressing indicated shRNAs. (F) Light micrographs show representative WM793 cell colonies that were formed in 3D collagen and fibrin after 16-d assay.
(TIF)

Figure S5 **Silencing of MT3-MMP does not have any notable effect on Bowes cell invasion.** (A) MT1-MMP and MT3-MMP mRNA levels in Bowes cells transfected with control siRNA (Ctrl) or siRNAs targeting MT1-MMP (siMT1) or MT3-MMP (siMT3) were detected by qPCR (n = 3). (B) Cells transfected with the indicated siRNAs were allowed to invade 3D fibrin and type I collagen for 5 d. Quantitative results are expressed as the number of invasive foci per microscopic field (n = 3). (C) Light micrographs of fibrin and collagen cross-sections visualize the invasion of Bowes cells transfected with the indicated siRNAs. White dotted lines mark the surface of the matrix. (D) Cells transfected with the indicated siRNAs were embedded in 3D collagen type I gels as single cell suspension. Light micrographs show the cells after 5-d assay (n = 3).
(TIF)
