# Supplementary figures and images for: Membrane-Type-3 Matrix Metalloproteinase (MT3-MMP) Functions as a Matrix Composition-Dependent Effector of Melanoma Cell Invasion
Source: PLoS One. 2011 Dec 2;6(12):e28325. doi: 10.1371/journal.pone.0028325 (PMC3229567; doi:10.1371/journal.pone.0028325)

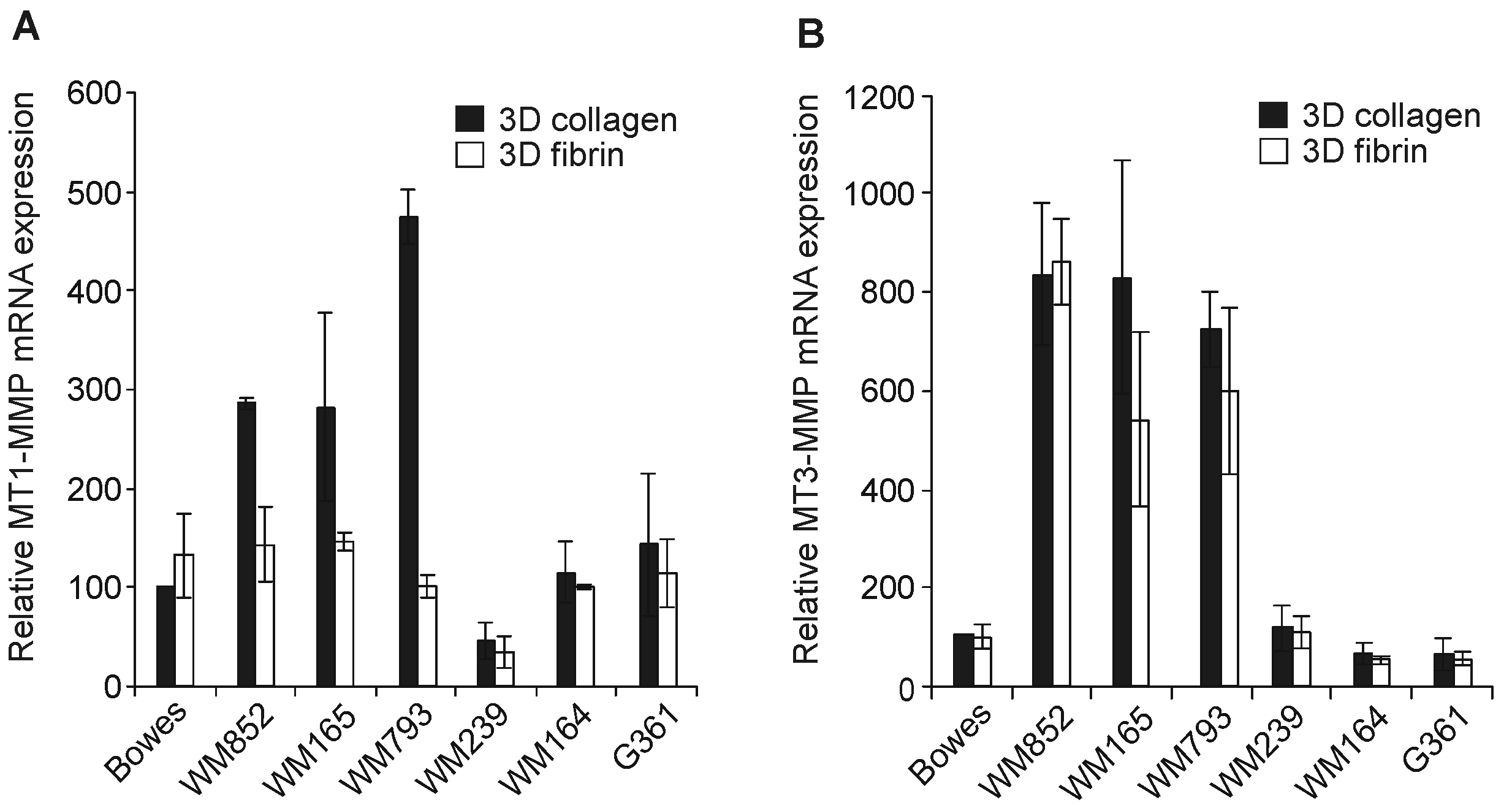

Supplement: Figure S1 — MT1-MMP and MT3-MMP expression in melanoma cells cultured within 3D collagen and fibrin. Relative values of average MT1-MMP (A) and MT3-MMP (B) mRNA expression in indicated melanoma cell lines cultured inside 3D collagen and fibrin gels for 48 h were analyzed by qPCR. MT1-MMP and MT3-MMP mRNA expression in Bowes cells cultured in collagen were set to 100%, n = 3. (TIF) [file pone.0028325.s001.tif]

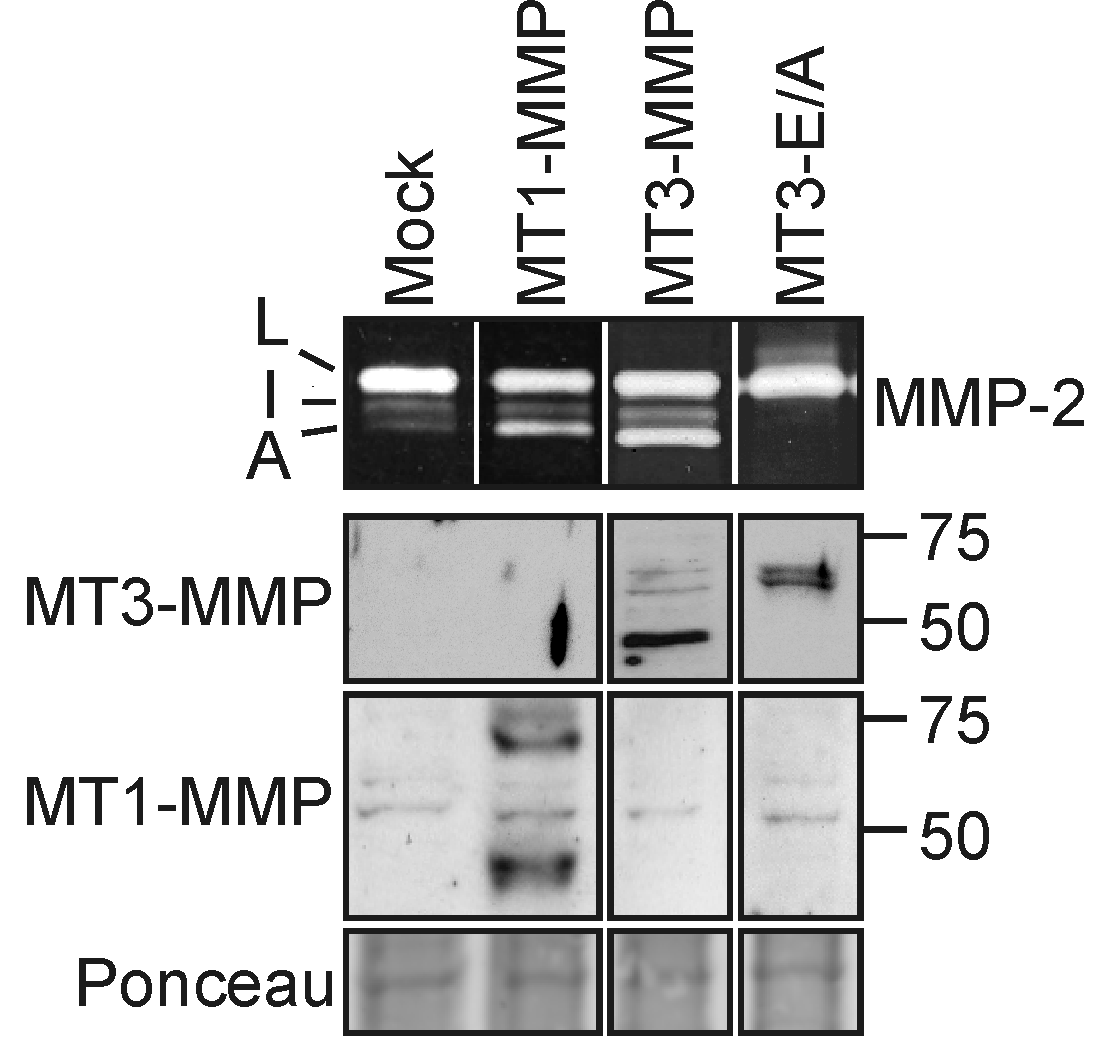

Supplement: Figure S2 — Catalytically inactive MT3-MMP does not activate MMP-2. Gelatin zymogram shows MT1-MMP-mediated MMP-2 activation by COS-1 cells expressing MT1-MMP, MT3-MMP or MT3E/A. MMP-2: latent (L), intermediate (I) and active (A). After harvesting of conditioned media, the cell lysates were subjected to immunoblotting for MT3-MMP and MT1-MMP. (TIF) [file pone.0028325.s002.tif]

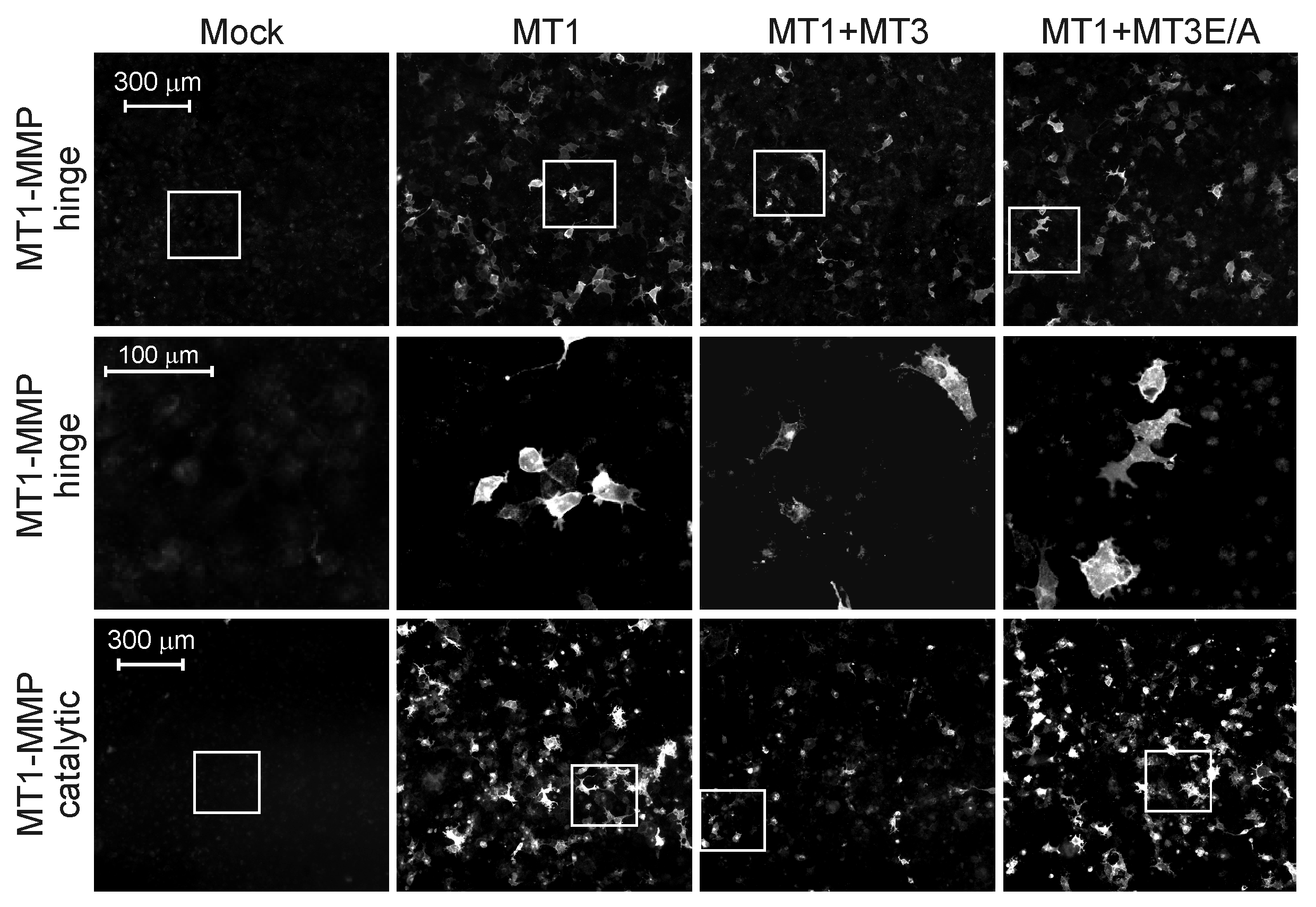

Supplement: Figure S3 — MT3-MMP reduces MT1-MMP on the cell surface. COS-1 cells transiently expressing MT1-MMP or MT1-MMP in combination with MT3-MMP or MT3E/A were fixed and stained with antibodies against the hinge domain (RP1) and catalytic domain (LEM2-15/8) of MT1-MMP. Magnified areas indicated by white boxes are presented below the each image for RP1 staining, and on figure 5E for LEM2-15/8 staining. (TIF) [file pone.0028325.s003.tif]

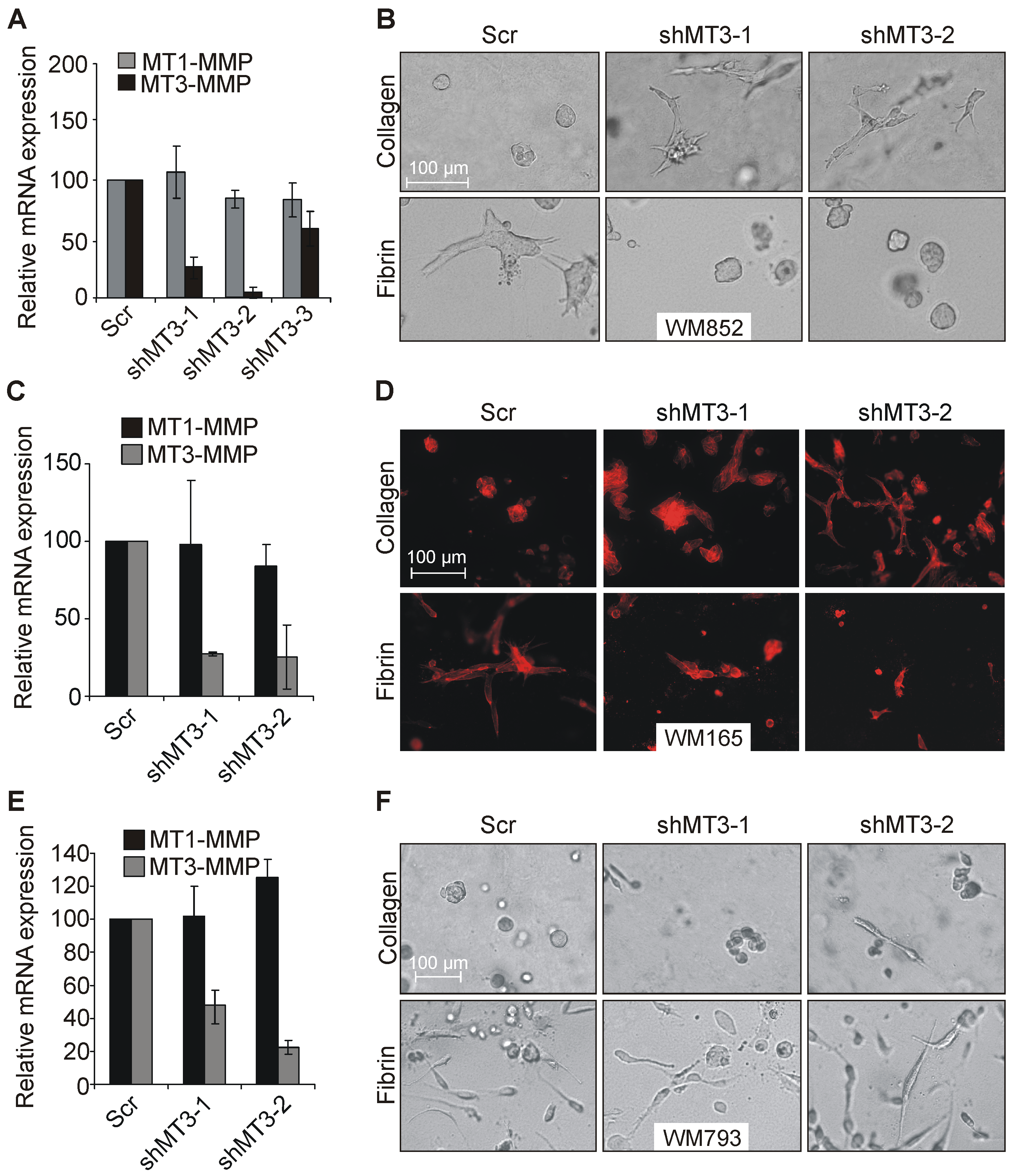

Supplement: Figure S4 — MT3-MMP modulates collagen and fibrin invasion of three different melanoma cell lines. (A) Average MT1-MMP and MT3-MMP mRNA expression in the WM852 cell pools expressing shRNA against MT3-MMP (shMT3-1, shMT3-2 and shMT3-3) relative to cells expressing scrambled shRNA (Scr). (B) WM852 cells stably expressing indicated shRNAs were embedded in 3D collagen type I or fibrin gels as single cell suspension. Light micrographs show representative cell colonies that were formed after 7-d assay. (C) Average MT-MMP mRNA expression in the WM165 cells expressing indicated shRNAs. (D) WM165 cells expressing indicated shRNAs were cultured inside 3D collagen type I and fibrin for 14 d, after which cultures were fixed and stained for filamentous actin. Quantifications of invasive areas are presented on figure 6B. (E) Average MT-MMP mRNA expression in the WM793 cells stably expressing indicated shRNAs. (F) Light micrographs show representative WM793 cell colonies that were formed in 3D collagen and fibrin after 16-d assay. (TIF) [file pone.0028325.s004.tif]

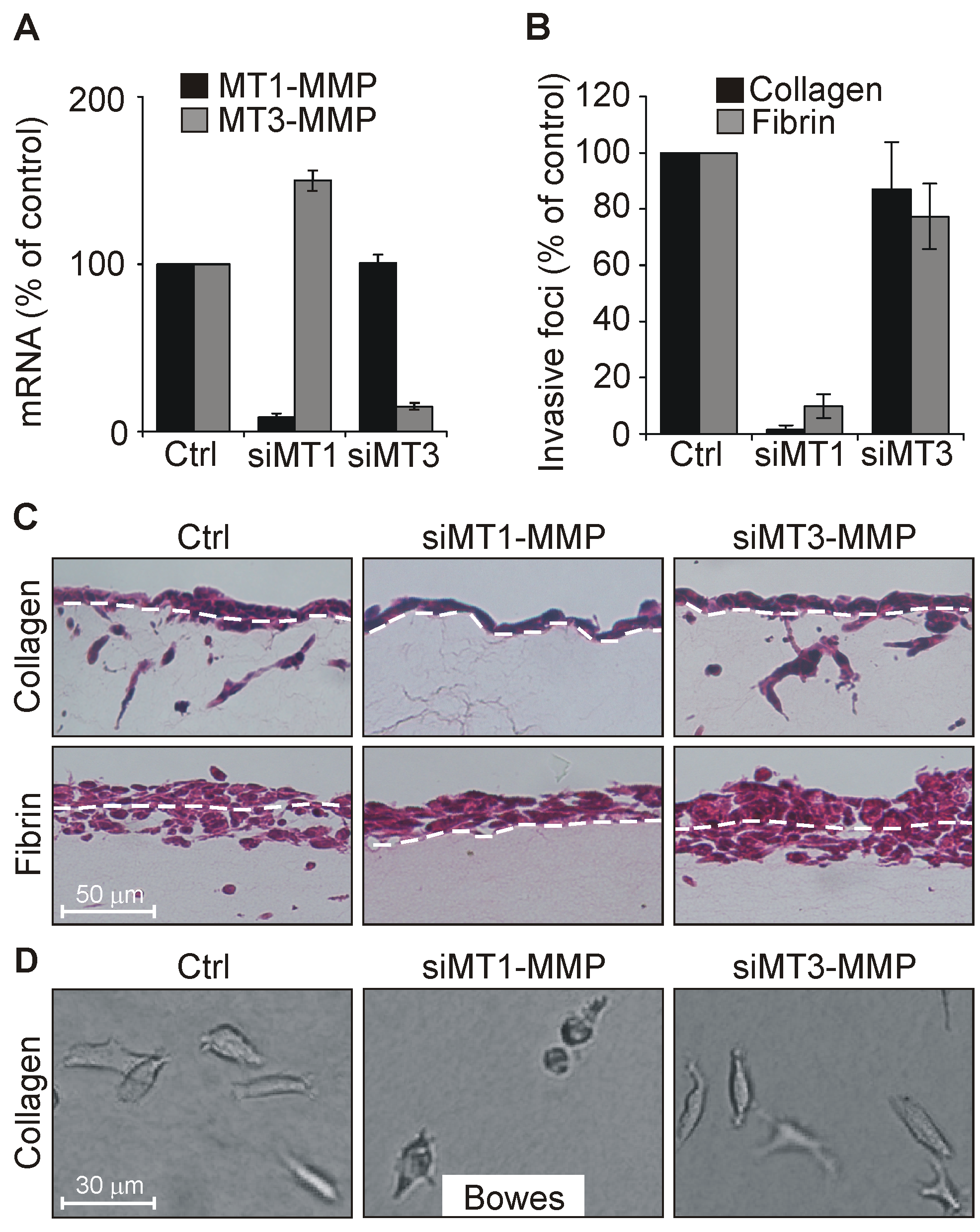

Supplement: Figure S5 — Silencing of MT3-MMP does not have any notable effect on Bowes cell invasion. (A) MT1-MMP and MT3-MMP mRNA levels in Bowes cells transfected with control siRNA (Ctrl) or siRNAs targeting MT1-MMP (siMT1) or MT3-MMP (siMT3) were detected by qPCR (n = 3). (B) Cells transfected with the indicated siRNAs were allowed to invade 3D fibrin and type I collagen for 5 d. Quantitative results are expressed as the number of invasive foci per microscopic field (n = 3). (C) Light micrographs of fibrin and collagen cross-sections visualize the invasion of Bowes cells transfected with the indicated siRNAs. White dotted lines mark the surface of the matrix. (D) Cells transfected with the indicated siRNAs were embedded in 3D collagen type I gels as single cell suspension. Light micrographs show the cells after 5-d assay (n = 3). (TIF) [file pone.0028325.s005.tif]
